# Energy harvesting based routing protocol for underwater sensor networks

**DOI:** 10.1371/journal.pone.0219459

**Published:** 2019-07-17

**Authors:** Adil Khan, Mukhtaj Khan, Sheeraz Ahmed, Mohd Amiruddin Abd Rahman, Mushtaq Khan

**Affiliations:** 1 Department of Computer Science, Abdul Wali Khan University, Mardan, KPK, Pakistan; 2 Department of Computer Science, Iqra National University, Peshawar, KPK, Pakistan; 3 Department of Physics, Faculty of Science, Universiti Putra Malaysia, UPM Serdang, Selangor, Malaysia; 4 Pakistan Ordinance Factory (POF), Wah Cantt, Punjab, Pakistan; RMIT University, AUSTRALIA

## Abstract

Underwater sensor networks (UWSNs) are ad-hoc networks which are deployed at rivers, seas and oceans to explore and monitor the phenomena such as pollution control, seismic activities and petroleum mining etc. The sensor nodes of UWSNs have limited charging capabilities. UWSNs networks are generally operated under two deployment mechanisms i.e localization and non-localization based. However, in both the mechanisms, balanced energy utilization is a challenging issue. Inefficient usage of energy significantly affects stability period, packet delivery ratio, end-to-end delay, path loss and throughput of a network. To efficiently utilize and harvest energy, this paper present a novel scheme called EH-ARCUN (Energy Harvesting Analytical approach towards Reliability with Cooperation for UWSNs) based on cooperation with energy harvesting. The scheme employs Amplify-and-Forward (AF) technique at relay nodes for data forwarding and Fixed Combining Ratio (FCR) technique at destination node to select accurate signal. The proposed technique selects relay nodes among its neighbor nodes based on harvested energy level. Most cooperation-based UWSN routing techniques do not exhibit energy harvesting mechanism at the relay nodes. EH-ARCUN deploys piezoelectric energy harvesting at relay nodes to improve the working capabilities of sensors in UWSNs. The proposed scheme is an extension of our previously implemented routing scheme called ARCUN for UWSNs. Performance of the proposed scheme is compared with ARCUN and RACE (Reliability and Adaptive Cooperation for efficient Underwater sensor Networks) schemes in term of stability period, packet delivery ratio, network throughput and path loss. Extensive simulation results show that EH-ARCUN performs better than both previous schemes in terms of the considered parameters.

## Introduction

Underwater Sensor Networks (UWSNs) are variation of Wireless Sensor Networks (WSNs) which operate under the surface of water. UWSNs observe different kinds of underwater phenomenon like seismic activities, pollution control and submarine movement etc. The UWSNs can be deployed in the form of one of the three models i.e, deployment of network with static sensor nodes, mobile sensor nodes and sensor nodes with Autonomous Underwater Vehicles (AUVs) [1]. However, UWSNs nodes deployed in any form of the model are operated with limited energy supply provided by non rechargeable batteries. In addition, the unbalanced utilization of energy greatly affects the performance of UWSNs. For this purpose, a number of protocols have been proposed in the literature to balance energy consumption and improve network lifetime [2–6].

In [2], the authors exploited the functionality of AUV to gather data from sensor nodes. The proposed technique relies on turning off the sensors at various locations during data gathering cycles in order to achieve balanced energy consumption, however this technique does not employ energy harvesting using cooperation at multi-relay communication. [3], discussed wireless sensor networks for balanced energy consumption. The main focus of this work is on the control of transmission power of sensor nodes with reduction in size of a data packet. [4], proposed a localization scheme; where each sensor node knows the exact location of its destination. Cooperation-based communication is opted when the source nodes have updated their routing tables based on Sound to Noise Ratio (SNR) of its neighboring relay nodes. In underwater data communication, due to low SNR and high path loss, the source node may not communicate directly with the destination. In this scenario, source node opts for the best set of relay nodes based on SNR and gain. These relay nodes cooperatively send data towards the destination as proposed in our scheme, however the proposed scheme cannot be considered for a network with mobile sensor nodes. Moreover the authors have merely derived an equation for energy and did not employ it in their mathematical model. [5], proposed a mechanism for efficient selection of a node as a cluster head in cluster based sensor network, however in this technique more energy is consumed at different cluster heads which cumulatively affect the network lifetime. [6], stressed a need for energy efficiency which mainly relies on AUVs. When AUVs are closer to any set of sensor nodes, the nodes forward their data to AUVs and then go into power-off mode. However this technique also does not employ energy harvesting at the relay nodes.

In all the aforementioned techniques, balanced energy consumption is achieved either by powering off the sensor nodes when the nodes are not sensing any data or, by the use of AUVs. Furthermore these models ignore energy harvesting mechanisms to improve network lifetime.

For long lasting operation of a network, several energy harvesting techniques have been proposed for wireless terrestrial sensor networks [7]. In [7], authors categorized energy harvesting schemes into two categories i.e (1): energy harvesting using solar energy and (2): energy harvesting using piezoelectric nanogenerators. The solar energy mechanism is not applicable in an underwater environment however, piezoelectric energy harvesting mechanism is used for biomedical, industrial, military and drug delivery systems. The piezoelectric technique used in wireless terrestrial sensor networks harvest energy from the wind. In [8], authors proposed a scheme based on radio signals for energy harvesting and in [9], the authors proposed a scheme for energy harvesting based on Received Signal Strength (RSS) of a radio signal from a source node. The radio signals have high absorption rate in an underwater environment and cannot be applied directly in an underwater sensor networks. In [10], the authors proposed solar and wind energy harvesting techniques in a terrestrial sensor networks, however this technique can be applied for terrestrial sensor networks but is not feasible to be deployed in an underwater environment.

Piezoelectric energy harvesting is the most feasible technique for underwater wireless sensor networks [11]. This technique can generate fair amount of voltage by applying pressure of water current on piezoelectric material which can generate electricity which in turn extend battery operation for UWSNs nodes. With piezoelectric energy harvesting, UWSNs sensor nodes are now capable to harvest energy from hydrophones to charge its batteries [11].

In cooperation based communication technique, source sensor node send data to the relay sensor node and destination sensor node simultaneously on two physically independent paths. Amplify and forward (AF) technique [12] is incorporated in the relay node to regenerate, amplify and forward the signal received from a source node. Destination node receive data from two sources simultaneously and apply fixed ratio combining technique [13] to analyze and select the most accurate signal out of the two sources. For reliable and efficient communication in UWSNs, many cooperation based communication protocols have been suggested in the current literature [14–17] and in [18, 19]. For example, [18] deployed a single relay node for cooperation however a single relay node quickly exhaust energy which decreases a network lifetime. In [19], the authors proposed RACE for cooperation, however it used normal sensor nodes as relay nodes which significantly affect the overall network lifetime.

In this paper we have proposed a novel Energy Harvesting Analytical approach towards Reliability with Cooperation for Underwater WSNs (EH-ARCUN). In this technique, source node select two relay sensor nodes as a working set. The proposed technique utilize two relay nodes for cooperation based communication. Amplify and forward technique is applied at relay nodes to regenerate and amplify a received data signal. The piezoelectric energy harvesting technique is applied on the relay nodes to increase a network lifetime.

Main contributions of this paper are summarized as follows:

- Piezoelectric energy harvesting based Analytical approach towards Reliability with Cooperation for Underwater WSNs scheme is proposed. In this scheme each energy harvesting relay sensor node decide when to switch between energy harvesting mode and data forwarding mode based on residual energy and channel condition.
- Capability of cooperation based communication is exploited in terms of incorporating energy harvesting capability in relay nodes as well as avoiding direct communication path between the source and destination pair of nodes.
- Through simulation, the feasibility of deploying piezoelectric energy as a source for energy harvesting in the relay sensor nodes is shown.

The performance of the proposed technique is compared with cooperation based communication techniques i.e ARCUN [18] and RACE [19] (Reliability and Adaptive Cooperation for Efficient Underwater Sensor Networks) in terms of stability period, packet delivery ratio, end-to-end delay, path loss and throughput of a network. The simulation results show significant performance improvement in terms of considered parameters.

Rest of the paper is organized as follows, Section II outlines related work about energy harvesting techniques and types of cooperation based communication routing protocols in UWSNs and terrestrial sensor networks. Section III lays out proposed protocol mechanism. Section IV present simulation results. Finally, section V concludes the paper and points out some future work.

## Related work

In this section, we present the related works. The related works are categorized into: (i) Cooperation based communication in UWSNs and (ii) Non cooperation based communication in UWSNs.

## 1 Cooperation based communication in UWSNs

[18] proposed ARCUN to improve network lifetime however, this scheme send data directly towards destination without considering euclidian distance. As a result of this direct transmission, source node consume more energy which consequently decrease the lifetime of whole network. Authors in [19] proposed a scheme which employed cooperation at physical layer to save energy consumption of network. However, the proposed protocol does not consider special relay nodes. All the cooperation based communication is performed by normal sensor nodes which quickly depletes energy for the whole network.

In [16], authors used amplify and forward technique at relay nodes and Fixed Ratio Combining technique (FRC) technique at destination node to increase the efficient use of energy and balance the data transmission load in a network. This technique does not employ energy harvesting enabled relay nodes, as a result the technique puts extra data forwarding load on the relay nodes, resulting in decreased network life time. Furthermore authors did not specify medium access control mechanism used in this scheme. In [14], hydrocast protocol is improved by employing cooperative technique of communication with intelligent placement of sensor nodes. Calculated deployment of sensor nodes ensures precise data gathering of observed field of interest. This technique does not consider node localization problem. Furthermore, in order to precisely place nodes at specific underwater regions, nodes should be tied with buoys through wires. In [20] authors proposed a mechanism based on graph theory, in this technique source node select a set of nodes in arbitrary direction which can avoid holes in its path towards destination. This protocol puts extra processing burden on each node for generating neighbouring nodes set. Deficiency of the scheme proposed in [21] lies at relay node which employ processing overhead involving error checking for each received data packet.

In [22], protocol is proposed to improve node localization in UWSNs network. In this protocol AUV nodes work as data providing nodes for ordinary sensor nodes. AUVs adjust their location according to its neighboring nodes. Although this scheme improves node localization problem in UWSNs but is not suitable for time sensitive application because ordinary sensor nodes will have to wait for AUV to come in sight of their transmission range. In [23] Depth and Energy Aware Cooperative routing protocol for UWSNs (DEAC) is proposed, in this scheme, sensor nodes depth is adjusted according to the sparsity of network thereby improving energy consumption. Although energy consumption is improved but adjusting node depth leads to dynamic topological change in the network which in turn can change density of some underwater region which will increase data forwarding load on sensor nodes in that region.

In [24] authors proposed Sink Mobility with Incremental Cooperative routing protocol for UWSNs (SMIC), this scheme utilize movement of sinks in order to efficiently cover monitored sensed area. Mobility of sinks is governed according to the network density. This scheme can only improve packet delivery ratio for the sensor nodes closer to the surface of the water. Furthermore, each sensor node will have to calculate its distance from sink node because of the dynamic movement of sink node, this will put extra processing load on each sensor node resulting in quick depletion of energy.

## 2 Non cooperation based communication in UWSNs

In [25] authors discussed and analyzed use of piezoelectric energy generators for UWSNs. This scheme does not mention number of energy harvesting relay nodes which are deployed with corresponding deployment cost of such relay nodes.

The protocol proposed in [26] use two kinds of algorithms, first is efficient routing algorithm: Using the optimum transmission range for the optimum energy consumption in the whole network creating static trees-route from underwater sensor nodes to sink. Second, data balancing transmission algorithm: underwater sensor nodes taking the decision for data transmission, that is based on the energy level of the successor nodes whether it is to be one hop or multi hop.

In [27] the achieved parameter is balanced energy consumption per node, which is the primary reason for an extended network lifetime. Proposed technique is applied in sparsely 2D environment by presenting two type of schemes for balanced energy consumption: first scheme analyze the data transmission of the network with mooring mechanism to find out unbalanced energy consumption, and the second scheme try to balance energy in dense and shallow water. The protocol Switch between single and multiple hop transmission which take time resulting in increased end-to-end delay.

In [28], energy driven protocol is proposed which divide the initial energy of each sensor node into segments known as energy levels. For data transmission, node is selected with high energy level. Each node has different energy level as a result, selection of node is based upon high energy level of any specific node. The protocol avoids energy depletion and improves network lifetime. Network is deployed with static configuration resulting in higher end-to-end delay.

In [29], authors have given solution for coverage hole avoidance by coverage repair algorithm and balanced energy consumption technique. In dense network, transmission range of each sensor node is excessively overlapped with each other, authors have taken advantage of nodes excessive overlapping to remove coverage hole problem. Authors have given solution to energy hole by shifting nodes from coverage region to energy hole region and these nodes are restricted for coverage hole formation. This scheme affects packet delivery ratio, prolong network life time, energy consumption and throughput. End to end delay is increased because of the time spent on removing coverage hole in network. In this scheme, the mobility of nodes is compulsory.

In [30] Multi hop Underwater Acoustic Local Area Networks (MUA-LANs), the network life time is prolonged with the help of optimum path of relay node and efficient energy consumption. In this paper three phases are presented, Network discovery, Relay path determination and association phase. In network discovery phase a node discover the network by broadcasting the hello packet to its neighbouring nodes. In relay path determination phase: authors deployed path lifetime and energy efficiency relay algorithm to select the relay node with optimum path to maximize the network lifetime by balancing energy consumption. In case two paths have equal lifetime, than this technique selects those paths which have maximum energy efficiency. Energy consumption is major issue in MUA-LAN, it is mostly caused by the transmission distances and traffic loads of different nodes. In association phase authors discuss forwarding with the help of MAC address tables, in this phase multi hop relay forwarding can be easily realized. Because of the heavy computation involved at each source node for selection of relay nodes, this scheme suffers from heavy end-to-end delay towards sink nodes.

Enhancement achieved in [31] is packet delivery ratio, energy consumption, throughput and end to end delay. In this paper authors have proposed an Ultrasonic Frog Calling Algorithm (UFCA) for 3D sparse environment, its functionality is to achieve effective energy utilization in severe under water environment. Every sensor node has its own transmission radius and every node stores residual energy value. Nodes with low residual energy and with high depth values are put to sleep mode. Nodes incorporate their location information within each packet. Because of the sleep mode, some of the sensor nodes will not sense data so this scheme can not be applied for real time monitoring of underwater phenomena.

On the basis of literature survey, two protocols ARCUN and RACE are selected for the purpose of comparison with proposed scheme EH-ARCUN. We have assumed the energy model presented in [32], which gives energy consumption computation for *R*_*x*_, *T*_*x*_ and amplification process computation. For easy understanding, a comprehensive comparison of different UWSNs protocols discussed in this section are presented in Table 1. The protocols are compared in terms of novelty, limitation, routing mechanism, data forwarding and data combining techniques. Amplify and Forward (AF) technique is applied at relay nodes, diversity combining techniques, Fixed Ratio Combining (FRC) and Maximum Ratio Combining (MRC) are applied at destination node. Next section provide detail for the proposed scheme.

**Table 1:** Comparison of different routing schemes.

Protocol	Routing Mechanism	Data forwarding at relay node	Data combining technique at destination node	Novelty	Disadvantage
Co-improved Hydrocast [14]	Cooperative	AF	MRC	Introduction of greedy algorithms	High computation cost at source and relay nodes, increased end-to-end delay
Co-EEUWSN [16]	Cooperative	AF	FRC	Linear cooperative path model	More data forwarding nodes, increased delay
ARCUN [18]	Cooperative	AF	FRC	Cost function for link selection	Increased delay
RACE [19]	Cooperative	AF	MRC	Cooperative diversity with one antenna	Increased energy consumption
RBCRP [21]	Cooperative	AF	MRC	Bit Error Rate (BER) check at destination node, Mobile Sinks	High end-to-end delay because of mobile sinks
Co-MobiL [22]	Cooperative	AF	MRC	AUVs equipped with GPS	Increase in end-to-end delay due to wait time for AUVs
SMIC [24]	Cooperative	AF	MRC	Incremental cooperation	Low channel capacity

## EH-ARCUN: Energy harvesting in ARCUN

**Fig 1:**
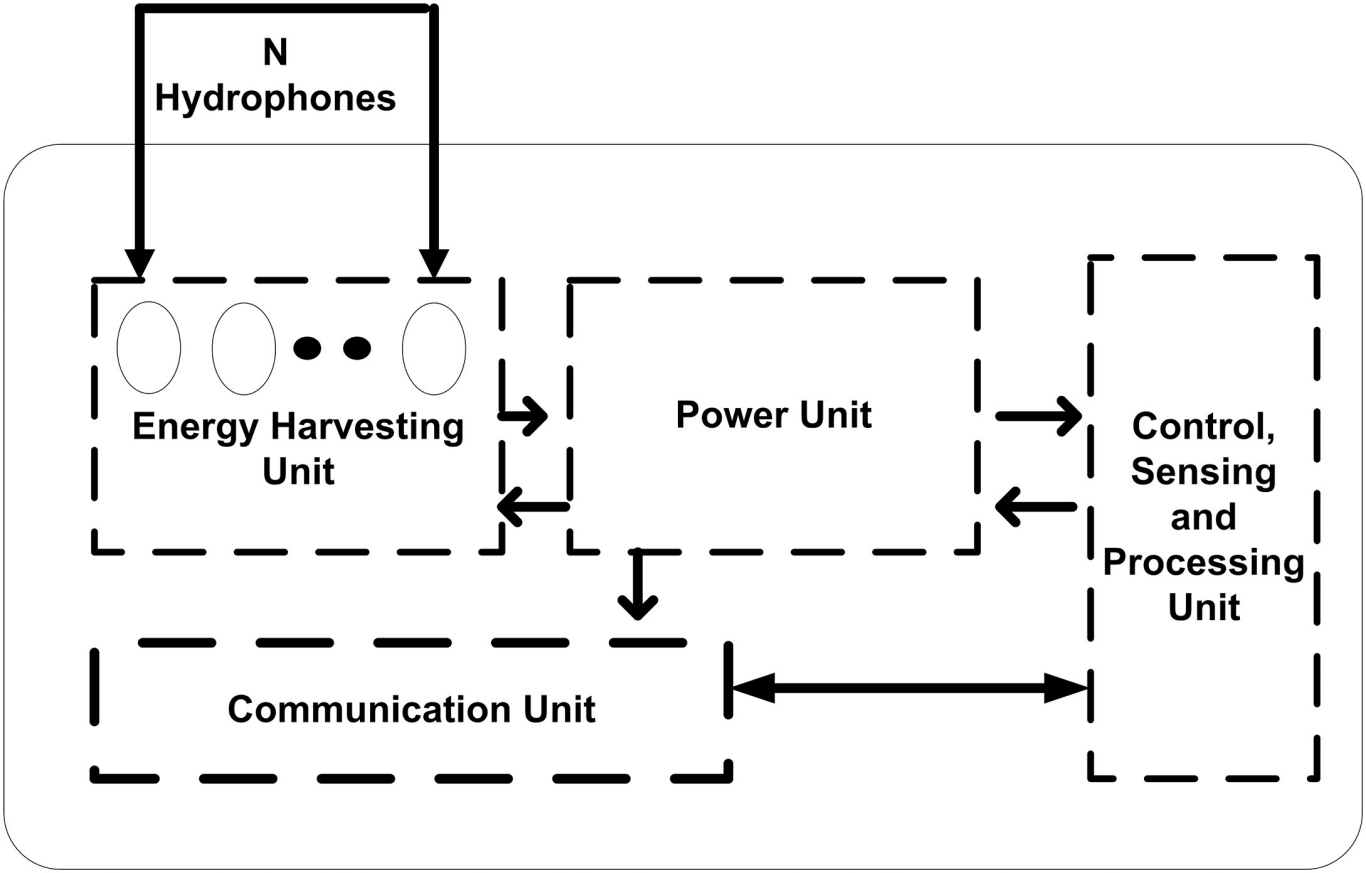
Remotely powered underwater acoustic sensor node [11].

In this section we propose EH-ARCUN scheme. The proposed scheme is based on source and destination nodes pair with two relay nodes for cooperation based communication. The relay nodes are special nodes with extra processing power to overhear the communication of a source node and forward the data of a source node with its own sensed data. The piezoelectric technique [11] is applied on relay nodes for energy harvesting purpose. The working of sensor nodes in piezoelectric energy harvesting is depicted in Fig 1.

In this research work, simulations are based considering underwater sensor nodes by iMOTE [33]. The dimension of these nodes ranges from 1 to 10 cm3. These sensors are designed particularly for acoustic signals with higher bandwidth. Until recently, the smallest commercially-available Poly Vinylidene Fluoride (PVDF) membrane hydrophone sensor [34] had a diameter of 0.5 mm, which is larger than the wavelength in water for frequencies above 3 MHz. An array of 2 to 3 hydrophones of.5mm size can very easily be installed on iMOTE chip with given specifications. By using array of hydrophones (i.e. 2 to 3 hydrophones) installed on small sized chip, harvesting unit can be developed which can take less space inside the overall sensor unit. This mechanism will ensure small battery size for relay sensor nodes. The Remotely Powered Cooperation based UWSN (RPCoU) technique is applied for cooperation based communication. Cooperation is employed with special energy harvesting relay nodes to enhance network lifetime in the proposed scheme. To increase reliability, we have employed technique of amplify and forward (AF) in the relay nodes for EH-ARCUN. Decode and Forward (DF) scheme can be employed at relay node, but DF scheme is more suitable for terrestrial WSNs.

**Fig 2:**
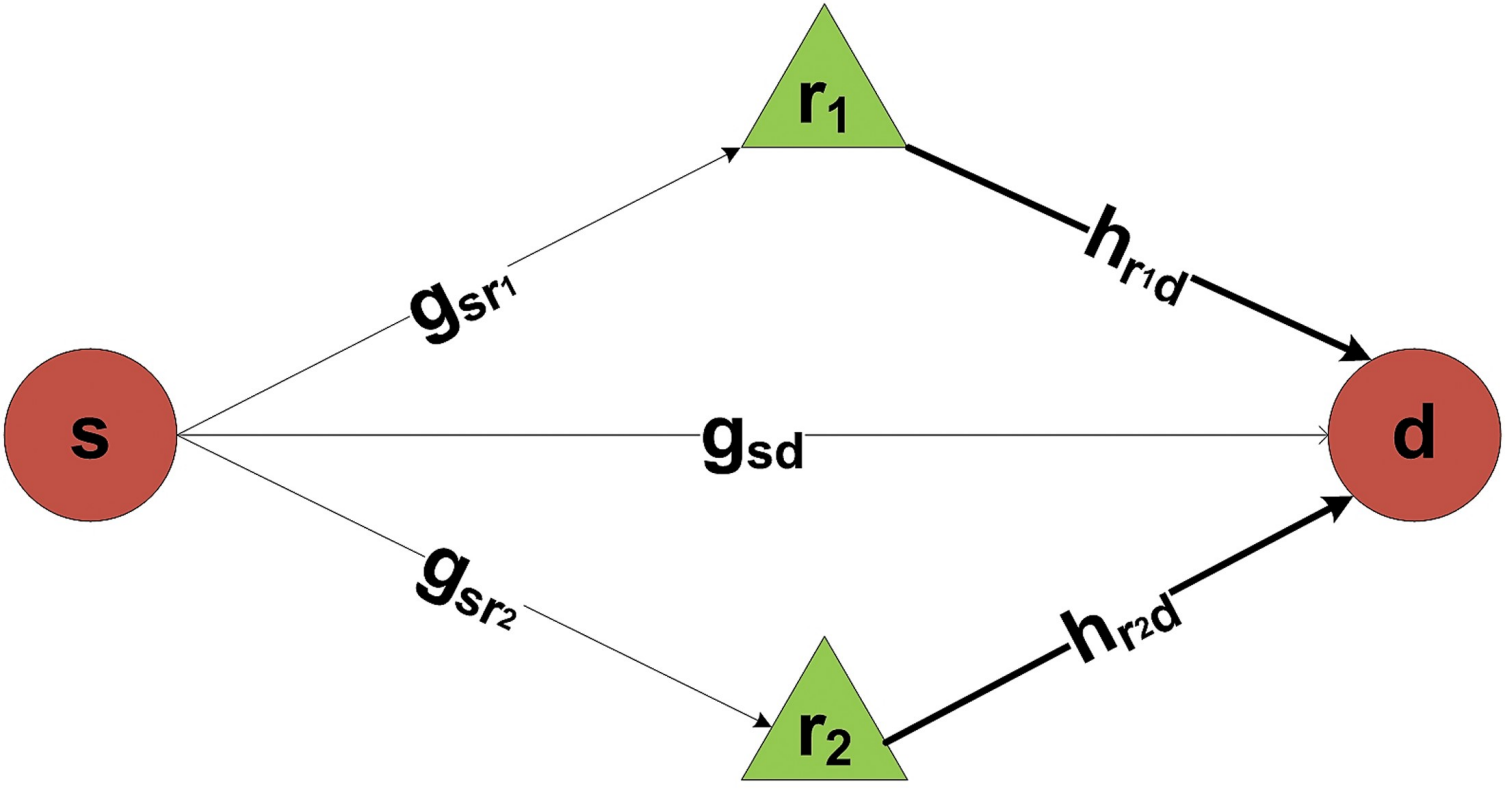
EH-ARCUN: One source and destination nodes pair with 2 energy harvesting relays.

In order to design the proposed scheme we assumed that 5% of 125 total nodes are selected as energy harvested relay nodes at three depth levels namely,low, medium and high respectively. This assumption ensures that at least two relay nodes are available for any source and destination pair as depicted in Fig 2.

### Channel attenuation

We have used Monterey-Miami Parabolic Equation model (MMPE) [25] to predict path loss and attenuation of signal from source node to relay and from relay to destination node instead of Thorps Attenuation Model. Both the techniques are the attenuation models for the underwater environment. Thorps model is being regularly utilized by all the researchers working in this domain and is an abstract type model. In order to have a deep insight into the attenuation parameters and a different approach, we considered MMPE model. This model is derived from Fourier analysis. The sound stress is considered in small segmented increments in transmission range and depth of sensor nodes, building coordinated two dimensional grid. It considers varying signal strength and diversity of sound signal for prediction, using a real time propagation loss calculation. The authors demonstrated that minute variations in water depth and inter gap between sensor nodes can cause large variations in propagation loss calculations as a result of deep water wave’s diverse motion which impacts underwater signaling (more details can be found in [14]). Propagation loss from source to relay node and from relay node to destination node for cooperative communication can be calculated using MMPE as given in Eqs (1), (2) and (3) respectively [35]:

$$\begin{array}{c} PL\left(t_{1}\right)=m\left(f,s,d_{S},d_{R}\right)+w\left(t\right)+N\left(f\right) \end{array}$$ (1)

$$\begin{array}{c} PL\left(t_{2}\right)=m\left(f,s,d_{R},d_{D}\right)+w\left(t\right)+N\left(f\right) \end{array}$$ (2)

where:

$$\begin{array}{c} N\left(f\right)=N_{t}\left(f\right)+N_{s}\left(f\right)+N_{w}\left(f\right)+N_{th}\left(f\right) \end{array}$$ (3)

and, *PL*(*t*_1_): propagation loss from source node to relay node and *PL*(*t*_2_): propagation loss from relay node to destination node. m()is propagation loss exclusive of arbitrary and cyclic components, obtained from degeneration of MMPE data. *f* is frequency of transmitted acoustic signals (in kHz), *d*_*S*_ is sender node depth (in meters), *d*_*R*_ is receiver node depth (in meters), *d*_*D*_ is destination node depth (in meters),*s* is Euclidean distance between nodes (in meters), *w*(*t*) is periodic function to approximate signal loss due to wave movement and *N*(*f*) is the aggregation of underwater turbulence, shipping, wave and thermal noises as given in (3).

m() function is calculated using channel coefficients *A*_*n*_. As wave signal strength varies significantly in underwater communications so linear equations are not applicable to predict path loss of signal, consequently non linear equation must be applied to provide close approximation for varied weakness of signal.

According to common law of physics waves travel through particles present in communication mediums. In underwater communications acoustic signal creates force which gives circular movement to particles. Radius of circular movements decreases with increase in underwater depth and is considered significant up to 50 meters of depth. Length of the radius depends on acoustic signal strength and amplitude of signal. According to MMPE model w() function given in Eqs (4) and (5) respectively give prediction of signal loss due to acoustic signal movement which can be modeled as [35]:

$$\begin{array}{c} w\left(t\right)=h\left(l_{w},d_{R},t,h_{w},T_{w}\right)*e\left(t,T_{w}\right) \end{array}$$ (4)

$$\begin{array}{c} w\left(t\right)=h\left(l_{w},d_{D},t,h_{w},T_{w}\right)*e\left(t,T_{w}\right) \end{array}$$ (5)

Where *h*() is scale factor function, *l*_*w*_ is ocean wave length (meters), *d*_*R*_ is depth of the relay node (meters), *d*_*D*_ is depth of the destination node (meters), *h*_*w*_ is amplitude of signal (meters), *T*_*w*_ is periodic length of signal (seconds) and *e*() is function for ambient wave noises.

Effects on acoustic signals due to the nature of signal and ambient noises are calculated on receiving relay and destination nodes.In our communication model multiple relay nodes are cooperatively deployed for forwarding received signal towards destination node. If the number of forwarding relays increases then ambient noise follows Gaussian distribution and can be modeled for source, relay and destination nodes respectively as in Eqs (6) and (7) respectively [35]:

$$\begin{array}{c} e\left(\right)=20\left(d\left(d_{S},d_{R}\right)*s_{max}\right)*R_{N} \end{array}$$ (6)

$$\begin{array}{c} e\left(\right)=20\left(d\left(d_{R},d_{D}\right)*s_{max}\right)*R_{N} \end{array}$$ (7)

where *e*() is ambient noise function, d(*d*_*S*_, *d*_*R*_) is distance between the source and relay nodes (in meters), (*d*_*R*_, *d*_*D*_) is distance between the relay and destination nodes (in meters), *s*_*max*_ is maximum transmission range (in meters) and *R*_*N*_ is random number from a Gaussian distribution.

### Energy harvesting in relays

For energy harvested relay nodes R as depicted in Fig 2, source level is an important factor to be considered, it not only determines the amount of energy which can be harvested but also the quality of harvested energy in terms of signal strength. Source level of acoustic signal can be calculated as in Eq (8) [11]:

$$\begin{array}{c} SL=170.8+10*log_{10}\left(P_{elec}\right)+10log_{10}\left(\eta \right)+DI \end{array}$$ (8)

Where *η* is conversion efficiency, P is signal power and DI is directional intensity. As depicted in Fig 2, h represents complex channel coefficients which leads to attenuation of signal. For energy harvesting attenuation level of signal must be considered and can be calculated as in Eq (9) [11]:

$$\begin{array}{c} AL=20*log_{10}\left(R\right)+a\left(f\right)*\left(R\right) \end{array}$$ (9)

Where R is transmission range of source node S. It may be noted that the greater the transmission range of a node, the greater is attenuation level of signal with a(f) representing attenuation over frequency. By calculating signal strength and attenuation factor, we can easily calculate a received signal level strength by using an equation as in Eq (10) [11]:

$$\begin{array}{c} RL=SL-AL \end{array}$$ (10)

Pressure p necessary for energy harvesting can be calculated as given in Eq (11) [11]:

$$\begin{array}{c} P=10^{\frac{RL}{20}} \end{array}$$ (11)

For energy efficiency *η* received voltage sensitivity (RVS) can be calculated as in Eq (12) [11]:

$$\begin{array}{c} RVS=20log_{10}\left(M\right) \end{array}$$ (12)

where M is voltage sensitivity. Using Eqs (11) and (12) we can calculate induced voltage gained as in (13) [11]:

$$\begin{array}{c} V_{ind}=\left(10^{\frac{RL}{20}}\right)*\left(10^{\frac{RVS}{20}}\right) \end{array}$$ (13)

maximum power available through n hydrophones is calculated as in Eq (14) [11]:

$$\begin{array}{c} P_{availble}=\frac{{\left(nV_{ind}\right)}^{2}}{4*nR_{p}}=\frac{n*V_{ind}^{2}}{4*R_{p}} \end{array}$$ (14)

Where *R*_*p*_ is energy harvesting hydrophone. Putting the value of Eq (11) in Eq (14) we get an actual harvested power for relay nodes as given in Eq (15) [11]:

$$\begin{array}{c} P_{harv}=\frac{0.7*n*10^{\frac{\left(RL+RVS\right)}{20}}}{4*R_{p}} \end{array}$$ (15)

### Relay selection based on harvested energy

It is assumed that a source node is surrounded by its immediate neighbouring nodes. A source node maintains a queue for all of its neighbouring nodes. An entry in a queue marks a neighbouring node satisfying *d*(*n*) < *d*(*s*). Where *n* identifies all the neighbouring nodes of a source node *s*. A relay node distinguish itself from a normal node by attaching a special value *max*(*P*_*h*_ *arv*) as in Eq (15) of available harvested energy in its Clear To Send (CTS) packet towards a source node. A source node selects 3 destination nodes, 2 nodes are selected as relay nodes and 1 node as a destination node. Before adding a neighbouring node, a source node checks the capacity of the data link with *C*(*f*, *l*) >= *R*, where *C* is in bits/seconds over some frequency and length between the link, and *R* is max data transmission rate of a node.

Now, we consider one specific scenario in which we must answer question of “which relay out of set of relay(s) will perform data forwarding?” and “which one will perform energy harvesting?”. To answer these two questions, in this paper we have developed maximum energy value for each relay node which must be maintained. Each relay *R*_*i*_ stores in its local queue, constant value for minimum energy that can be harvested by hydrophones denoted by *P*_*harv*_ as in in Eq (15). Each relay has independent choice of either to harvest energy or forward received data from source node. It will only relay data if,

$$\begin{array}{c} P_{harv}\geq £ \end{array}$$ (16)

Where *£* in Eq (16) is constant minimum attainable harvested energy value, otherwise it will execute duty cycle for energy harvesting. This can be modeled as given in Eq (17) [18]:

$$\begin{array}{c} E_{re}\left(S\right)\leq E_{re}\left(R_{i}\right)*P_{harv}\;where\;P_{harv}\geq £ \end{array}$$ (17)

Relay node amplifies received data from source node before forwarding it towards destination, as we employed amplify and forward (AF) scheme in our protocol, this amplification can be represented by factor *ψ* i.e $y_{r_{i}d_{i}}=\psi \left(y_{s_{i}r_{i}}\right)$. if $P_{s_{i}r_{i}}$ and $P_{r_{i}d_{i}}$ are transmission powers from source and relay respectively, then *ψ* can be written as modeled in Eq (18) [18]:

$$\begin{array}{c} \psi =\sqrt{\frac{P_{r_{i}d_{i}}+P_{harv}}{P_{s_{i}r_{i}}|T_{d(s_{i}r_{i})}{|}^{2}+N{\left(f\right)}^{2}}} \end{array}$$ (18)

As power P is considered as energy gained in specific time Δt, Eq (18) can be rewritten as Eq (19) [18]:

$$\begin{array}{c} \psi =\sqrt{\frac{E_{r}+P_{harv}}{E_{s}|T_{d(s_{i}r_{i})}{|}^{2}+N{\left(f\right)}^{2}*\Delta t}} \end{array}$$ (19)

If we consider channel fading which is generally considered to be not dependant on time then Eq (19) can be rewritten as Eq (20) [18]:

$$\begin{array}{c} \psi =\sqrt{\frac{E_{r}+P_{harv}}{E_{s}|T_{d(s_{i}r_{i})}{|}^{2}+N_{s_{i}r_{i}}{\left(f\right)}^{2}}} \end{array}$$ (20)

### Cooperation implementation

Eq (15) can be used to calculate the amount of harvested energy at relay node. We have assumed that relay node operates in two modes: one is energy harvesting mode and the other one is data forwarding mode. When in data forwarding mode, data is received at relay node and destination node from source node. Relay node applies amplify and forward technique. Data received at relay node and destination node simultaneously can be modeled as in Eqs (21) and (22) [18]:

$$\begin{array}{c} y_{s_{i}r_{i}}=P_{s_{i}r_{i}}g_{s_{i}r_{i}}x_{s_{i}}+N_{s_{i}r_{i}}\left(f\right) \end{array}$$ (21)

$$\begin{array}{c} y_{s_{i}d_{i}}=P_{s_{i}d_{i}}g_{s_{i}d_{i}}x_{s_{i}}+N_{s_{i}d_{i}}\left(f\right) \end{array}$$ (22)

Where $x_{s_{i}}$ is data received from source node, $P_{s_{i}r_{i}}$ and $P_{s_{i}d_{i}}$ is power of signal from source node to relay node and destination node respectively, *g* represents channel coefficients with added underwater noise N. Data received by relay node is forwarded towards destination node using amplify and forward technique and can be modeled as in Eq (23) [18]:

$$\begin{array}{c} y_{r_{i}d_{i}}=P_{r_{i}d_{i}}h_{r_{i}d_{i}}x_{s_{i}}^{\prime }+N_{r_{i}d_{i}}\left(f\right) \end{array}$$ (23)

$P_{r_{i}d_{i}}$ is power of signal from relay node to destination node, where $P_{s_{i}r_{i}}=P_{r_{i}d_{i}}$ if attenuation is low and relay receives signal without any considerable error, otherwise data packet may be rejected by relay node. It may be noted that in our scheme two relay nodes forward data from same source node towards destination as depicted in Fig 2.

### Combining strategy at destination node

Each destination node D employs assortment integration mechanism to put together signals received from two relay nodes. In fixed rate combining (FRC) technique, instead of aggregating received signals, they are assigned weights based on fixed ratio. This ratio represents the average channel efficiency and reflects the choice of destination node on which relay channels should be considered for received signal and consequently the received data. As discussed in MMPE model every path in underwater communication suffers from different channel losses due to ambient noises thus assigning weights to different relay channels is feasible choice. In case of two relay nodes, FRC can be expressed as [18]:

$$\begin{array}{c} y_{d}=k_{1}y_{r_{1}d}+k_{2}y_{r_{2}d} \end{array}$$ (24)

In Eq (24) *y*_*d*_ represent an integrated signal received from two relay nodes. *k*_1_, *k*_2_ represents weights assigned to two physically independent data links of relay nodes. These weights represents aggregate channel condition for individual source and relay node pair. Different parameters affect channel condition for example, salinity level, distance, sound to noise ratio etc. By combining these weights, destination node ensures to select optimal relay node for a received data signal. Considering power of these links with corresponding channel complexities and using weights of relay nodes according to their distances from source and destination nodes, there ratios can be calculated as in Eq (25) [18]:

$$\begin{array}{c} \frac{k_{2}}{k_{1}}=\frac{\sqrt{P_{2}}h_{r_{2}d}}{\sqrt{P_{1}}h_{r_{1}d}} \end{array}$$ (25)

## Simulation results of EH-ARCUN

We use MATLAB r2007B to simulate 3D underwater sensor networks. A network layer is developed to simulate EH-ARCUN, ARCUN and RACE. We use (Data Collection Oriented Medium Access Control) DCO-MAC protocol [36]. In this MAC protocol, the sink broadcasts the Ready To Receive (RTR) packet. The sensor nodes sends the Available To Send (ATS) packets to the sinks. This MAC protocol avoids collision by assigning random time slots to each sensor node. In our simulations, we set the data transmission rate as 1 packet every 1 second, which can help to effectively demonstrate the energy harvesting efficiency of relay nodes. For the physical layer data transmission, we set the parameters similar to a commercial acoustic modem, LinkQuest UWM2000 [37]. The bit rate is 19k bps; the transmission range is 250 meters; and the energy consumption in sending mode, receiving mode and idle mode of sensor nodes are 2.0w, 0.8w and 8mw respectively. We set the packet size to 100 Bytes.

In all the simulation experiments described in this section, sensor nodes are randomly distributed in a 3D field of 600m×600m×600m. There are 3 static sinks with 125 nodes which are arbitrarily laid out in a network. The sinks are fixed at locations (100,100,0), (400,400,0) and (600,600,0) respectively. Each sink is separated by 200m from each other. Besides the sink, all other sensor nodes including the relay nodes are mobile as follows: they can move in horizontal two-dimensional space, i.e., in the xy-plane (which is the most common mobility pattern in underwater applications). Each source node randomly selects the 2 best energy harvesting relay nodes according to the SNR gain in its neighbouring node routing table. These relay nodes sends the data towards their destination node as depicted in Fig 2. The destination node performs FRC technique to select the best data signal out of 3 data signals received from 2 energy harvesting relay nodes and a data signal received directly from a source node. Destination node forward the data to the next higher region using cooperation of neighboring relay nodes until the data arrives at sink. For each simulation run, the results are averaged over 100 runs, with an arbitrarily produced topology in each run. The total simulation time for each run is 5000 seconds depicted as rounds in simulation graphs.

**Fig 3:**
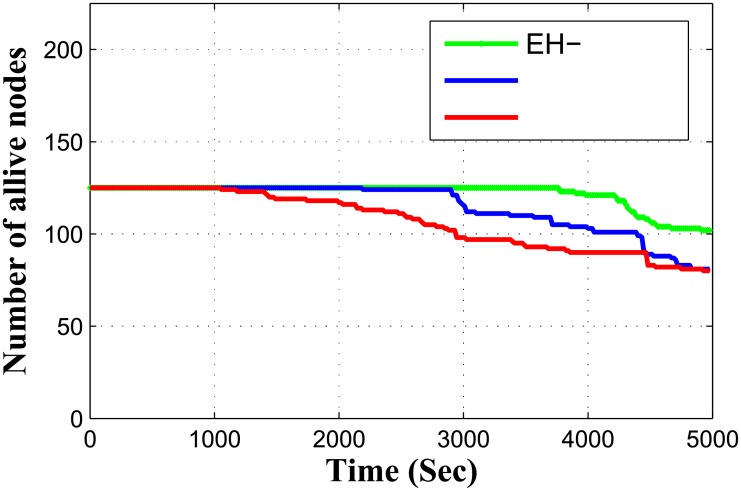
Stability period vs time.

In each round, sensor nodes select potential relays after identical intermission of time, nodes calculate their distance from their neighboring relay nodes. Presence of energy harvesting relay nodes with its threshold value for maintaining energy efficiency, makes EH-ARCUN scheme more efficient for data critical applications. The proposed scheme EH-ARCUN is being compared with RACE and ARCUN (existing techniques in literature) these schemes have already utilized AF technique, hence for fair comparison the proposed technique improvement can only be judged when the same parameters be taken into account. Fig 3 show that EH-ARCUN scheme improve the stability period of a network due to the integration of energy harvesting relay sensor nodes.

Simulations result in Fig 3, show that the first node in RACE dies after 1200 seconds, in ARCUN the first node dies after 3000 seconds and in our EH-ARCUN scheme first node dies after 4000 seconds which points towards improvement in stability of a network. In ARCUN relay nodes without energy harvesting capability are deployed, these nodes exhaust their energy quickly. Furthermore source node transmits data simultaneously on two physically independent communication channels i.e. towards relay node and destination node respectively. The destination node is located at maximum transmission range of source node which puts extra transmission load on source node. This phenomenon eventually becomes the cause of instability. RACE scheme does not consider any relay nodes, every node is homogeneous, employing cooperation technique for data forwarding, eventually reducing the stability period of a whole network.

**Fig 4:**
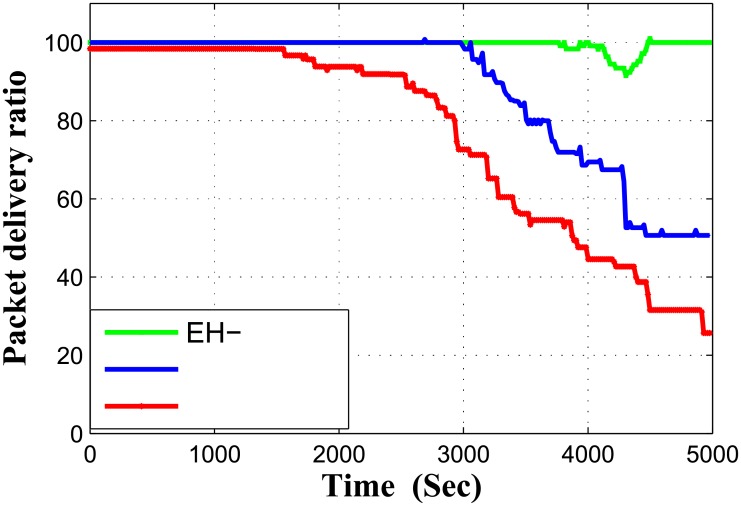
Packet delivery ratio vs time.

**Fig 5:**
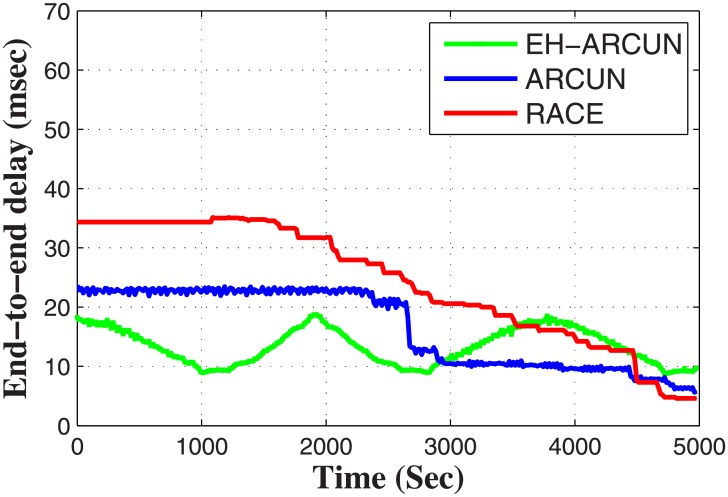
End-to-end delay vs time.

Packet Delivery Ratio (PDR) is shown in Fig 4. Packet delivery ratio is defined as number of packets received at sink as compared to actually transmitted data packets by sensor nodes. PDR of EH-ARCUN and ARCUN shows similar progression up till 3000 seconds, where significant drop of PDR can be noted for ARCUN. Because of amplify and forward schemes deployed in ARCUN, at some point signals are not received in their acceptable quality and amplification is not possible, because of that packets are normally dropped by relay nodes. In RACE PDR drop is very visible in contrast to EH-ARCUN and ARCUN, RACE does not consider relay node mechanism, although it employs cooperation based communication technique but because of the decrease in inter arrival time of packets, packet collision increases which affects delivery ratio. At the end of the simulation the sudden drop of packet delivery ratio for RACE scheme indicates another important factor that all sensor nodes have exhausted their residual energy and there is no alive node left. End-to-end delay is plotted as shown in Fig 5.

It indicates an increasing trend at the end of simulation time for EH-ARCUN. It is comparatively larger than ARCUN and RACE scheme. The main reason for increase in the delay of our scheme is time taken by relay nodes to harvest energy. More duty cycles are spend on energy conversion efficiency to harvest relay nodes when network become sparse during passage of time. Initial delay of our proposed scheme is much less than ARCUN and RACE schemes. The decrease in delay for ARCUN and RACE schemes at the end of simulation indicates that all the nodes die out and delay time will approach zero in case if we increase the simulation time,it means that EH-ARCUN is more stable as compared to ARCUN and RACE schemes.

**Fig 6:**
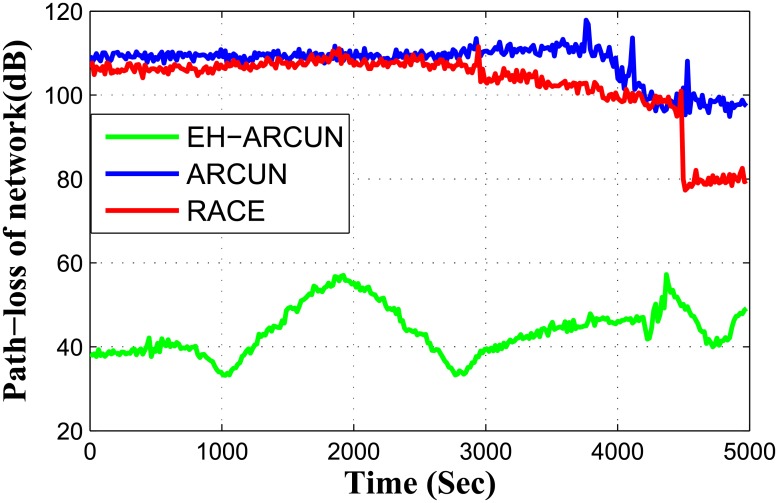
Path loss vs time.

Path loss is plotted in Fig 6. Path loss indicates signal strength with respect to transmission distance between source and destination nodes. As Fig 6 indicates, path loss of our scheme EH-ARCUN shows great improvement over ARCUN and RACE schemes.

**Fig 7:**
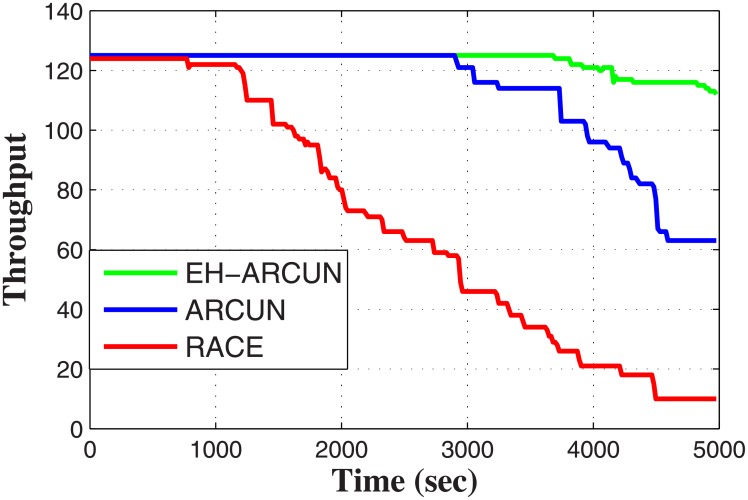
Network throughput vs time.

As stability period improves, it directly impacts network throughput as shown in Fig 7. Both ARCUN and RACE follow thorps attenuation model. We have incorporated Monterey-Miami-parabolic equation (MMPE) model for attenuation in EH-ARCUN. MMPE considers transmission range of every source node when it forwards data towards relay node. In addition, MMPE also considers signal architecture which include, amplitude and wave length of the acoustic signal. In EH-ARCUN scheme, each source node forward data to a relay node with minimum distance, larger distance between source and relay nodes greatly impact path loss of a signal.

More number of alive nodes means more data packets are arriving at sink in a specific time period. Energy harvesting capable relay nodes play crucial role in providing better throughput for the whole network, even though in sparse conditions every UWSN throughput will gradually decrease but still our scheme demonstrates much better performance as compared to ARCUN and RACE.

It can be observed from Figs 3 to 7 that the plots of the proposed model is constantly fluctuating. The main reason of the fluctuation is the energy harvesting process. As per the functionality of the harvesting, the proposed scheme cannot supply energy constantly to any relay node but it is done periodically or need based when energy level fall below a threshold value. In RACE and ARCUN, there is no energy harvesting mechanism and the plots show a non-variant behaviour whereas, in the proposed scheme, harvesting is being done at various instances which is the major cause of fluctuations that exhibit in all the plots of the proposed scheme weather it is packet delivery ratio, end-to-end delay or path loss.

## Conclusion

In UWSNs, inefficient energy consumption and feasible energy harvesting are challenging issues. These issues significantly affect the overall performance of a network. In this paper, we proposed a novel energy harvesting mechanism called EH-ARCUN based on our previous work. The proposed scheme utilized two relay nodes for cooperation based communication. Amplify and forward technique applied at relay nodes for strengthening and forwarding the signals. Fix combing ratio technique deployed at destination node to analyze and select accurate signal. The piezoelectric technique applied for energy harvesting purpose. Moreover, the proposed scheme used threshold value of minimum harvested energy which ensured relay nodes not to spend too much time in energy harvesting duty cycles, which leads to better stability period of network and efficient packet delivery ratio. The simulation results show that proposed scheme significantly improves overall performance of the UWSNs. In addition, the proposed scheme performs better than the existing representative works such as the ARCUN and RACE. It remains to be seen that how much energy each relay should spend on forwarding a data, because different relays would have harvested varied amount of energy and energy usage will be different for each relay node. Furthermore for future research direction, we would like to develop local relay selection scheme for source nodes, which will provide more efficient data forwarding choice for source nodes.
